# Management of Stone Disease in the Spina Bifida Patient

**DOI:** 10.1007/s11934-025-01300-5

**Published:** 2025-10-27

**Authors:** Meghan F. Davis, Kyle L. Yu, Arun K. Srinivasan

**Affiliations:** 1 Division of Urology, Department of Pediatric Surgery, The Children’s Hospital of Philadelphia, Philadelphia, PA USA; 2 Sidney Kimmel Medical College, Thomas Jefferson University, Philadelphia, USA

## Abstract

**Purpose of the Review:**

This review provides a detailed overview of the specifics of presentation, diagnosis, and management of upper and lower urinary tract stone disease for individuals with spina bifida.

**Recent Findings:**

Recent studies highlight the significant burden of stone disease for spina bifida patients.

**Summary:**

Individuals with spina bifida require lifelong urologic care. They are more likely to have stone disease and have complications from management. This is a particularly salient issue for patients who have undergone bladder augmentation. Given the frequency and severity of these issues, it is critical that urologists be familiar with the nuances of stone disease in this population. Further strategies are needed to decrease the morbidity associated with stone disease amongst individuals with spina bifida.

## Introduction

Stone disease is a common issue facing individuals with spina bifida and the clinicians that care for them. Unfortunately, individuals with spina bifida are more likely to have stone disease, require more invasive treatment, and experience complications from management. It is critical that urologists be familiar with the nuances of stone disease in this population. This review highlights the specifics of presentation, diagnosis, and management of upper and lower urinary tract stone disease for individuals with spina bifida, highlighting the most recent publications on this topic. While there is a sizeable literature addressing stone disease in patients with neurogenic bladder (NGB), much of this focuses on patients with spinal cord injury. While there are similarities to those with spina bifida, there are also fundamental differences given the congenital nature of spina bifida, and so, this review highlights studies addressing patients with spina bifida and extrapolates from research on spinal cord injury and all causes of NGB where specific evidence is lacking. Specific considerations for surgical planning and post-operative care are summarized in Table 1.

**Table 1:** Considerations and recommendations for clinical care

Pre-Operative	Obtain full surgical history and records, including knowledge of orthopedic or neurosurgical hardware Consider cross sectional imaging Treat pre-operative urine cultures to decrease infectious complications Consider pre-operative CBC and INR if performing PCNL
Intra-Operative	Allocate appropriate equipment and time for safe positioning Tailor antibiotics to local resistance profiles Limit operative times Minimize intra-renal pressures Send stone material for analysis
Post-Operative	Educate care teams on increased risk of post-operative complications to ensure timely diagnosis and treatment Consider electing a higher level of care for post-operative monitoring Ensure adequate drainage
Routine and follow-up care	Routinely discuss stone risk factors and symptoms with patients Perform metabolic work-up for first time stone formers Obtain periodic imaging to monitor for stone disease and recurrence Implement a high-volume irrigation protocol amongst those with bladder augments and provide support to optimize adherence Consider prophylactic antibiotics to prevent recurrence of infectious stones

## Burden of Urolithiasis

Stone disease is associated with significant burden for individuals with spina bifida. It is well documented that patients with spina bifida are at an increased risk for lower urinary tract stones, particularly after bladder augmentation. In the 10 years after bladder augmentation surgery, one-third of patients will undergo bladder stone surgery [1]. At the same time, there is also an increased risk of upper tract stones for patients with spina bifida. In a study focused on the pediatric population, 4.0% of patients with spinal dysraphism had upper tract stones compared to 0.24% of patients with normal spines [2].

Stone disease affects an already vulnerable subset of the spina bifida population. Amongst patients with spina bifida, those who have stones are more likely to have other comorbidities, which likely both increase their risk of stone disease and complicate treatment [3]. Compared to patients without spina bifida, patients with spina bifida and stones were more likely to be younger and have public insurance [4].

Treatment is also more complex and complication ridden for patients with spina bifida. Multiple procedures and more invasive procedures are more often to be required to address stone disease [2, 4]. Patients with spina bifida were more likely to have post-operative complications following stone surgery, including urinary tract infection (UTI), respiratory insufficiency, and prolonged mechanical ventilation [4].

The higher frequency of stone disease, the need for multiple and more invasive procedures, and the increased frequency of post-operative complications has significant implications on the healthcare system. Compared to spina bifida patients without stones, patients with spina bifida and stone disease have over twice as many healthcare encounters, as demonstrated in a recent analysis of statewide data from California [3]. Increased utilization persisted for those with stone disease even after controlling for comorbidities and sociodemographic factors [3].

### Clinical Presentation and Diagnosis

Upper and lower urinary tract stones may present with symptoms of UTI, abdominal or flank pain, difficulty with catheterization, or gross hematuria. However, stones are often found incidentally [5–7]. This is likely because urinary tract imaging is being more frequently performed as part of ongoing urologic care in patients with spina bifida.

Studies of bladder stone composition favor a predominance of infectious stones [7–9], however some studies report a mix of non-infectious and infectious stones with no clear majority [6, 10, 11]. Similarly, studies of upper tract stone composition are mixed with some reporting mainly calcium stones [5], while others report infectious stones predominate by varying degrees [7, 12]. Ultimately, this reflects the many risk factors associated with stone formation in patients with spina bifida, including recurrent UTIs and metabolic disturbances, and underscores the importance of performing 24-hour urine testing and stone analysis to ensure appropriate subsequent management.

## Risk Factors

Of the various risk factors for urolithiasis, those most relevant to the spina bifida population are interdependent and include recurrent UTI, urinary stasis, catheterization, and urinary tract reconstruction.

Urinary tract reconstruction, while bringing certain benefits, is a risk factor for stone disease. Bowel augments secrete mucus that is a nidus for bladder stone formation [8]. Bladder augmentation using ileum or colon is correlated with an increased risk for stone formation compared to gastric tissue [8] or ureter [13], however there has been a shift away from augmentation with these tissues for other reasons. The specific augmentation configuration can also contribute to stone formation if oriented with an irregular shape that leads to stasis [13]. Similarly, a concomitant bladder neck procedure is associated with a greater risk of bladder stone formation, likely mediated by increased stasis [14].

Bladder augmentation is also associated with an increased risk of nephrolithiasis [5, 15]. Salama et al. (2021) found that the risk of nephrolithiasis was 1% annually each year after bladder augmentation in a study of 427 patients with spina bifida [5]. On multivariate analysis, patients who were older at augmentation and who developed bladder stones post-augmentation were more likely to develop nephrolithiasis. This may be because, in the setting of vesicoureteral reflux, mucus from the augmented bladder refluxes to the upper tract where it serves as a nidus for nephrolithiasis [5]. Patients on an effective irrigation regimen may have less mucus resulting in decreased risk of both bladder and renal stones. The association between the development of bladder stones and renal stones suggests another possible explanation: increased surveillance of the urinary tract following augmentation may result in more diagnoses [15].

Wheelchair use has been associated with an increased risk of urolithiasis amongst spina bifida patients post-augmentation in some studies [5, 15], but not others [6, 16]. Non weight bearing status could contribute to stone formation in two ways. First, wheelchair use and low physical activity may result in sediment collecting more readily in consistently dependent calyces or portions of the bladder [6]. Second, patients with spina bifida are more likely to have osteoporosis and fracture [17]. Decreased mobility because of fracture may lead to increased bone turnover and resultant hypercalciuria [17, 18]. Fractures are more than three times more common in children compared to adults (10.9/1000 vs. 2.9/1000 patient years) and occur more commonly in non-ambulatory patients (OR 9.8) [17]. Other etiologies for decreased mobility, such as immobility following major surgery, can also increase stone risk [17].

Other factors likely also play a role in stone formation. Shepard et al. (2017) identified that decreased serum chloride, a finding consistent with contraction alkalosis and dehydration, was associated with stone formation post-augmentation [16]. Consistent with this theory, Hamid et al. (2008) found significantly lower urine volumes and higher urine pH amongst stone formers post-augmentation [10]. Additionally, they found significantly lower urinary citrate and higher urinary calcium amongst stone formers. A study by Husmann (2016) evaluated 24-hour urine studies on recurrent bladder stone formers; hypocitraturia was found in 75% of cases, while low urine volume was found only in a minority of cases [11]. These studies highlight the various factors contributing to stone formation and the utility of 24-hour urine studies for individual patients.

## Evaluation and Monitoring

While there are no specific guidelines for monitoring for stone disease in this population, patients with spina bifida and resultant NGB should have regular upper tract monitoring for stone disease or other unfavorable upper tract changes. Close monitoring of patients with spina bifida for the development of stones is imperative and can potentially reduce the burden of subsequent treatment and potential associated complications.

Following diagnosis and treatment, it is prudent to perform a metabolic evaluation for first-time stone formers with spina bifida to decrease recurrence risk. Specific consideration can be given to long-term prophylactic antibiotics if there are recurrent infectious stones [19]. Regular follow-up imaging should be performed given a higher likelihood of recurrence.

Observation of renal stones in the spina bifida population has not been specifically described. Lane et al. (2019) described outcomes of renal stones for spinal cord injury patients in their institutional NGB database. Forty-one stone episodes occurred (mean size 4.9 mm). 75% were initially observed and, of these, 16% underwent treatment at a median of 44 months (IQR 19,67) while the remainder passed spontaneously or remained unchanged [20]. If observation is elected, interval monitoring and imaging is imperative.

## Surgical Management of Upper Tract Stones

### Technical Considerations

When surgical intervention is required for upper tract stones in a patient with spina bifida, there are some special considerations. Having up-to-date pre-operative imaging and full knowledge of the patient’s surgical history is critical in order to choose the most effective surgical approach. Hardware from neurosurgery or orthopedic surgery may obscure fluoroscopic visualization. Prior urologic surgery, particularly bladder neck reconstruction may make accessing the ureters retrograde difficult. Body habitus and anatomy may create challenges in positioning—while these can be overcome, one must ensure appropriate time and equipment available for safe positioning to avoid injuries. These same factors may make traditional anatomic landmarks less useful, particularly in percutaneous nephrolithotomy (PCNL), which may create challenges with access, necessitating alternative approaches or assistance from interventional radiology.

### Stone Free Rates

Several groups have reported on stone free rates (SFR) for PCNL in the spina bifida population. Chaundry (2017) reported on 15 patients with spina bifida who underwent PCNL at a single institution, 78% of whom had a prior bladder augmentation [21]. Following PCNL, 60% were stone-free based on post-operative fluoroscopy or post-operative imaging. The lower-than-typical SFR was attributed to abnormal skeletal anatomy and prior surgeries including ventriculoperitoneal shunt, spinal hardware, and bladder augmentation, which can make identifying landmarks more challenging particularly during fluoroscopic procedures [21]. The researchers assessed the relationship between scoliosis angle and SFR in hopes of quantifying the relationship between the SFR and altered skeletal anatomy, however this relationship was not significant, likely indicating that the challenges created by differences in anatomy can most often be overcome and cannot be simplified into one value [21].

Mitchell et al. (2018) reported on a cohort study of 13 patients with spina bifida compared to 50 patients without spina bifida managed with PCNL. After initial PCNL, the SFR was 46% for those with spina bifida compared to 82% for those without spina bifida [12]. It should be noted that nearly half of the spina bifida patients required two punctures compared to less than a third of controls, and even still, a significantly lower SFR was achieved. The multiple punctures may increase the SFR but may also bring additional complications [22]—which will be discussed further in the next section. Other studies of patients with various causes for NGB have reported similar SFRs of 60–62% [22, 23].

While there have not been reports focusing on outcomes of ureteroscopy (URS) in patients with spina bifida, SFR following URS for patients with NGB are known to be lower compared to controls (63% vs. 86.6%) [24]. More recently, Parikh et al. 2025 reported on outcomes of URS for pediatric patients with NGB (of which 5% had spina bifida) [25]. The SFR amongst those with NGB was 61%; while not significantly different from the control cohort, this is low compared to the overall SFR for pediatric URS (58–100%) [25, 26].

### Complications

Aside from a lower SFR, complications following stone surgery are more frequent in those with spina bifida. This is driven by multiple factors. The comorbidities in the spina bifida population undergoing PCNL are different than the general population. In the Mitchell et al. study, 35% of spina bifida patients undergoing PCNL had restrictive lung disease and 29% were obese compared to 0% and 6% in the control group respectively [12]. Surgical factors such as multiple punctures and increased operative time also play a role [12, 22].

Increased likelihood of post-operative UTI following upper tract stone procedures has been demonstrated across several studies with estimates ranging from 8.2% to 24% [4, 25, 27]. Wang et al. found a 12.4% risk of UTI following extracorporeal shock wave lithotripsy (ESWL), PCNL, URS and stent placement compared to 6% amongst those without spina bifida in a nationwide analysis [4]. In the same study, sepsis was the second most common complication, experienced by 7% [4]. Estimates of post-operative UTI are as high as 24% in a French nationwide study of over 5,000 patients undergoing ESWL, URS, PCNL, and open or laparoscopic upper tract stone removal procedures [27]. Given the higher rate of post-operative UTI across these studies, treating pre-operative urine cultures, tailoring antibiotics to local resistance patterns, and limiting intra-renal pressures and operative time are key.

Aside from infectious complications, bleeding complications are more common amongst spina bifida patients undergoing upper tract stone surgeries (OR 2.3) [4], and post-operative transfusion is more common amongst spina bifida patients undergoing PCNL compared to patients without spina bifida (11.8% vs. 1.6%) [12], and so, pre-op lab evaluation with a complete blood count should be considered. Further, patients with spina bifida were more likely to have other complications including pneumonia (OR 1.5), prolonged mechanical ventilation (OR 2.1), pulmonary embolism (OR 3.0), acute renal failure (OR 1.9), and cardiac issues (OR 2.4) even after adjusting for co-variates [4]. This is an important part of counseling for these procedures. All clinicians involved in the care of these patients must be aware of these additional risks, so that complications are promptly identified and managed.

Patients with spina bifida are more likely to have longer length of stays (3.7 vs. 1.9 days) [4] and post-operative intensive care unit (ICU) admission—with nearly 30% needing ICU admission compared to 10% in one study focusing on PCNLs [12]. Given this, clinicians should have a lower threshold to elect for higher levels of post-operative monitoring.

Retreatment rates are also significantly higher. In a French nationwide study comparing patients with neurologic conditions (including spinal dysraphism, paraplegia, and multiple sclerosis), reoperation rates were 19.3%, 25.5% and 28.8% at 12, 18 and 24 months following an upper tract stone procedure, compared to 5.4%, 7.7% and 9.3% for non-neurological patients at the same intervals in a French nationwide study [27]. The researchers did not differentiate between incomplete treatment and retreatment, so it is likely that these percentages reflect some proportion of incomplete treatment as well as recurrence.

## Surgical Management of Bladder Stones

### Technical Considerations

Surgical management of bladder stones may be performed via cystoscopy per urethra or catheterizable channel, percutaneous cystolithotomy (PCCL), or open cystolithotomy. Channel caliber can limit the size of usable tools, making PCCL or open cystolithotomy preferrable for large stones [6], however with the advent of access sheaths with suction, removing broken fragments has become less time consuming and facilitates better stone clearance. At the same time, there is concern that approaches utilizing a catheterizable channel can compromise the continence mechanism, however the frequency of this is not widely reported. While moderately sized stones could be removed via a channel after fragmentation, PCCL can also be preferable in this setting to facilitate removal of all fragments—as it is our experience that residual, seemingly small stones left behind after fragmentation serve as a nidus for recurrence, necessitating repeat procedures. Following PCCL, it is important to ensure adequate drainage so that the percutaneous access site can heal—either by placing a catheter through the catheterizable channel or placing a separate suprapubic catheter.

### Surgical Outcomes

SFR following PCCL are estimated to be 66–71% [21, 28]. Chaundry (2017) assessed for patient and surgical factors associated with SFR, however the association with stone burden, body mass index, and operative time was not significant [21]. This is significantly lower than the SFR following PCCL for patients without NGB [29]. The lower SFR is likely multifactorial and driven by many of the same factors that were discussed above related to lower SFR following PCNL.

### Complications

Few studies have reported on complications following bladder stone surgery amongst individuals with spina bifida, but the limited evidence suggests a lower complication rate related to surgeries for bladder stones as opposed to upper tract stones. Four of seventeen patients who underwent PCCL experienced complications, including urine leak, gross hematuria, and mental status changes [21]. In another study of seven patients with augmented bladders who together underwent nine PCCLs, one experienced a UTI and urine leak [28]. In a larger study of 112 spinal cord injury patients who primarily underwent transurethral procedures, 17% experienced a complication, of which more than half experienced sepsis [30].

Recurrence is a significant risk for patients with bladder stones post-augmentation and appears greater in the first 2 years post-procedure [6]. Analysis by Szymanski et al. did not demonstrate any differences in recurrence risk based on technique or fragmentation [6]. While surgical approach was not associated with a reduction in recurrence, initiating a bladder irrigation protocol is an effective way to decrease stone risk. Hensle et al. demonstrated that initiating an irrigation protocol decreased subsequent bladder stone risk from 42% to 7% [9]. This study focused on 91 patients with mixed causes for reconstruction with various types of intestinal segments. Of note, before initiating the irrigation protocol, the stone risk was highest for spina bifida patients (67%) compared to those who underwent augmentation for exstrophy (25%) or posterior urethral valves (10%). The prescribed irrigation regimen was 240 ml of saline twice per week and gentamicin irrigation once per week. Husmann (2016) utilized a protocol of daily irrigations with 240mL compared to 60 or 120mL to decrease bladder stone recurrence in a population of spina bifida patients with ileal and sigmoid augments [11]. In addition to the irrigation regimen, three months of prophylactic antibiotics were prescribed following the bladder stone surgery. This study demonstrated significantly fewer bladder stone recurrences in those utilizing the high-volume irrigations—approximately 10% in the high-volume group vs. 50% in the lower volume groups at 5 years [11]. While these regimens are beneficial, adherence to a strict irrigation schedule is challenging. A study of adult spina bifida patients at Indiana University found that none were strictly adhering (≥ 6 times per week) to an irrigation schedule [31]. While these regimens are beneficial, it is important to provide careful and clear counseling about the risk of bladder stones following augmentation and as well as the necessary support to facilitate compliance. In the Indiana study, higher adherence was associated with self-catheterization compared to catheterization performed by caregivers [31].

## Conclusions

Individuals with spina bifida require lifelong urologic care. Stone disease is a common issue facing these individuals and their care teams. Unfortunately, individuals with spina bifida more frequently have stone disease and complications from management. Given this, it is imperative that urologists be familiar with the nuances of stone disease in this population. Specific considerations and recommendations for surgical planning and post-operative management are summarized in Table 1. Further strategies are needed to decrease the incidence, complications, and financial burden of stones amongst individuals with spina bifida. Efforts to this end are made challenging by the relative rarity of spina bifida compared to other causes of NGB and the variability in stone disease, and hence the need to extrapolate data from other populations with NGB.

## Key References

- 3. VanderVeer-Harris, N., et al., Comorbid Urolithiasis Significantly Increases Utilization Across Healthcare Settings for Individuals With Spina Bifida. Urology, 2025. Analysis of the California Department of Health Care Access and Information database from 2005 to 2017 highlighting differences in utilization for spina bifida patients with stones and spina bifida patients without stones.
- 25. Parikh, Y., et al., Ureteroscopy for stone disease in pediatric patients with neurogenic bladder: A single institution case-control study. J Pediatr Urol, 2025. 21(1): p. 29–34. Evaluating outcomes and complications of 275 ureteroscopic procedures in pediatric patients.
- 27. Beraud, F., et al., Incidence and safety outcomes associated with active stone removal procedures (ASRP): a comparison between neurological and non-neurological patients using the French National Health Data Base. World J Urol, 2022. 40(7): p. 1821–1827. French national health database study comparing hospitalizations and complications for neurogenic (multiple sclerosis, spinal dysraphism, paraplegia, tetraplegia) and non-neurogenic populations.

## Data Availability

No datasets were generated or analysed during the current study.
